# Local Atomic Configuration in Pristine and A-Site Doped Silver Niobate Perovskite Antiferroelectrics

**DOI:** 10.34133/2022/9782343

**Published:** 2022-02-25

**Authors:** Jing Gao, Wei Li, Jue Liu, Qian Li, Jing-Feng Li

**Affiliations:** ^1^State Key Laboratory of New Ceramics and Fine Processing, School of Materials Science and Engineering, Tsinghua University, Beijing 100084, China; ^2^Neutron Scattering Division, Oak Ridge National Laboratory, Oak Ridge, Tennessee 37831, USA

## Abstract

Antiferroelectrics have attracted increasing research interests in recent years due to both their great potential in energy storage applications and intriguing structural characteristics. However, the links between the electrical properties and structural characteristics of distorted perovskite antiferroelectrics are yet to be fully deciphered. Here, we adopt local-structure methods to elucidate the nanoscale atomic structure of AgNbO_3_-based antiferroelectrics and their structural evolution upon La doping. The local structural features including interatomic distance distributions and atomic displacements have been analyzed using neutron small-box pair distribution function (PDF) refinement in conjunction with large-box Reverse Monte Carlo modelling. Our results highlight the correlation of cation displacements in AgNbO_3_ and its disruption by the incorporation of La, apparently in corroboration with the observed anomalous dielectric properties. Spatial ordering of cation vacancies is observed in La-doped AgNbO_3_ samples, which coordinates with oxygen octahedral tilting to relieve lattice strain. These results provide renewed insights into the atomic structure and antiferroelectric phase instabilities of AgNbO_3_ and relevant perovskite materials, further lending versatile opportunities for enhancing their functionalities.

## 1. Introduction

The research and development of high efficiency energy storage materials is becoming a thriving research field. Compared to electrochemical energy storage systems such as batteries, dielectric capacitors are more favorable for applications which require high power densities, i.e., high charge/discharge speed [1, 2]. Antiferroelectrics with antiparallel dipoles exhibit a unique reversible antiferroelectric-ferroelectric phase transition, upon which electrical energy is rapidly stored and released [3]. AgNbO_3_ and its derivatives are emergent lead-free antiferroelectrics whose great potential in energy storage applications makes them promising candidates to substitute conventional lead-containing antiferroelectrics. The intriguing structural characteristics and high energy storage properties of AgNbO_3_-based antiferroelectrics have obtained growing research interest from both fundamental and practical aspects [4–7]. Undoubtedly, further clarifying the correlations between structure and physical property is not only of fundamental importance but reveals also essential information for material design.

Regardless of the high energy storage property achieved thus far, little is known about the role of their structural disorder and the link to physical properties. One significant feature of the structural distortion of AgNbO_3_ is oxygen octahedral tilting which occurs due to the relatively small size of Ag^+^ ion. At room temperature, the tilting follows a scheme of (*a*^−^*b*^−^*c*^−^)/(*a*^−^*b*^−^*c*^+^) in the Glazer notation [8], giving rise to a multiplied lattice of $\sqrt{2}a_{c}\times \sqrt{2}a_{c}\times 4a_{c}$ with respect to the cubic unit cell. Another characteristic of most interest is cation displacement, which forms antiparallel dipoles along the ±[110]_c_ direction [9]. In particular, previous studies revealed that the B-site Nb cation manifests additional local displacements correlated along the <001>_c_ direction, meaning that the resultant local displacement is along the <11 ± l>_c_ direction. The octahedral chains with opposite <001>_c_ displacement are mixed spatially, and therefore, the cation displacement, on average, is along the <110>_c_ direction [10, 11]. Such displacement correlations produce sheets of diffuse scattering in electron diffraction patterns [12]. Despite the conclusions achieved thus far, the exact cation displacement, especially the size and distribution of the additional [001]_c_ displacement, is still elusive, and their effects on the dielectric responses remain poorly understood.

Three polymorphs, M_1_, M_2_, and M_3_ phases, with the orthorhombic *Pbcm* space group are generally assigned to the average structure of AgNbO_3_ within the temperature range below 626 K. This structure is centrosymmetric with antiferroelectric ordering [9], which however, contradicts the weak ferroelectricity experimentally observed in the M_1_-phased AgNbO_3_ [13, 14]. To solve this contradiction, Yashima et al. proposed the noncentrosymmetric *Pmc2_1_* model by which the antiparallel dipoles do not cancel each other completely, providing a compelling explanation for the weak ferroelectricity (ferrielectricity) [15]. Nevertheless, the subtle disparity between these two configurations renders the detection of the detailed structure of AgNbO_3_ rather arduous.

Chemical modification has proven an effective method to alter the phase stability of AgNbO_3_-based antiferroelectrics. Among the doping strategies on either A-site or B-site, La-doped AgNbO_3_ is of particular interest because it demonstrates substantially enhanced antiferroelectric phase stability and energy storage density compared with pure AgNbO_3_ [16]. Previously, it has been qualitatively observed that the lattice distortion of AgNbO_3_ is suppressed upon La doping, as confirmed by the decreased distortion index of cation-oxygen bond length [16]. These observations, however, are summarized from the long-range crystallographic characteristic, whereas it remains unclear how the lattice structure is altered at the local scale.

Currently, a wide range of experimental results have demonstrated that functionalities of dielectric materials depend sensitively upon their fine structural characteristics [17, 18]. These characteristics, which are generally overlooked by conventional diffraction methods, usually exhibit some degree of deviation from the average periodicity [19, 20]. The aim of this work is to provide a comprehensive insight into the nanoscale atomic configuration of AgNbO_3_ with combined pair distribution functions (PDF) and the Reverse Monte Carlo (RMC) simulation method. Our results corroborate that the noncentrosymmetric *Pmc2_1_* space group provides a better description of the room-temperature structure of pure AgNbO_3_. Large atomic model is reasonably constructed using an RMC method, where the impact of La doping on local cation displacements is clarified. The spatial distribution of the <001>_c_ displacement of cations yields nanoscale correlated regions, which is disrupted by La doping. The disordered nanoscale correlations are further associated with the broadening of the temperature-dependent dielectric constant in La-doped AgNbO_3_.Our current work has depicted a holistic picture of the structure of AgNbO_3_ over multiple length scales and unravelled the generic mechanism responsible for the variation in the dielectric property, thus contributing to the design and characterization of relevant dielectric materials.

## 2. Results and Discussion

### 2.1. Local Structural Characteristics from PDF Refinements

**Figure 1:**
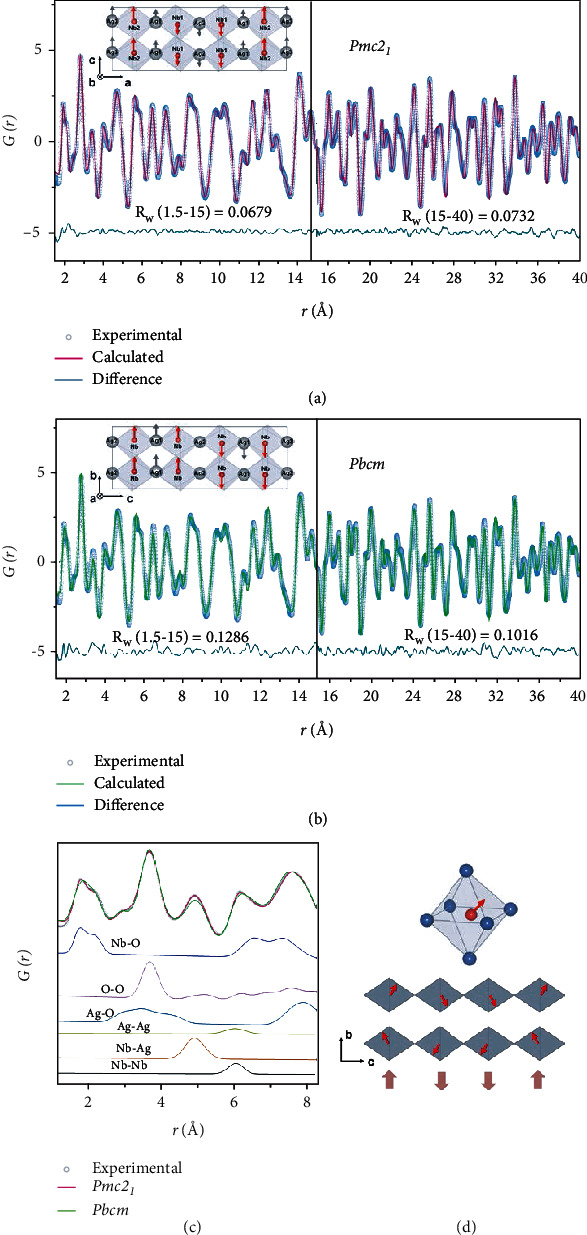
Room temperature PDFs of AgNbO_3_. (a, b) Experimental pattern fitted with Pmc21 and *Pbcm* space groups for the *r* = 1.5-15 Å and the 1-40 Å ranges. (c) Experimental *G*(*r*) with partial PDFs of atomic pairs shown below. (d) Schematics of the local Nb displacements along the <111>_c_ direction and their correlations which give rise to the average <110>_c_ antipolar ordering.

Pair distribution function (PDF) proves a powerful tool for local structure characterizations of a wide variety of perovskite-structured materials [21–24]. Defined in real space, PDF contains information of both Bragg diffraction and diffuse scattering, the latter of which is usually overlooked by conventional Rietveld refinements [25]. Figures 1(a) and 1(b) present the results of neutron PDF data for AN, where the calculated pattern is overlapped on the experimental data and a refinement is conducted on two *r*-regions of 1.5-15 Å and 15-40 Å. Note that 15 Å is a distance close to the dimension of one unit cell of AgNbO_3_, and thus, this dividing line is set to distinguish the local and intermediate range. The noncentrosymmetric *Pmc2_1_* and centrosymmetric *Pbcm* space groups are used as starting models for comparison. Overall, the experimental data are better fitted with the *Pmc2_1_* space group than *Pbcm* within both ranges. Over the distance of 1.5-15 Å, the refinement using *Pmc2_1_* improves the weighted residual *R*_*w*_ from 0.1286 to 0.0679. Similar difference, albeit less significant, is found for the 15-40 Å range. The fitting to the *Pmc2_1_* and *Pbcm* space groups yields a *R*_*w*_ factor of 0.0679 and 0.0732, respectively, indicating that the distinction between the two configurations fades out as the scale range increases.  Figure 1(c) provides a closer inspection of the low *r*-region of 1.5-5 Å, where the corresponding partial PDFs for specific atom pairs are presented below. The shoulder of Nb-O pairs indicates a bimodal distribution of Nb-O distances, which is primarily due to the off-center displacement of Nb ion along the <111>_c_ direction (Figure 1(d)). Particularly, the *Pmc2_1_* space group gives a satisfactory fit to the first nearest Nb-O pairs, indicating that this model is more likely to fit the structure of AN with the <001>_c_ displacement at the local scale. Such compatibility with the local structure originates from the noncentrosymmetric nature of *Pmc2_1_* space group which removes symmetry constraints for the additional component of displacement.

However, the PDF pattern of AN at 500 K shows a different trend with respect to those at 300 K. At this temperature, the *Pbcm* model provides a slightly better description than the *Pmc2_1_* model with *R*_*w*_~0.11-0.13 versus *R*_*w*_~0.14-0.15 (Figure S1). Refinements are also conducted for ANL4 where the data obtained at both 300 K and 500 K are considered (Figure S2). The *R*_*w*_ factor of the fitting with *Pmc2_1_* structure deteriorates with increasing temperature, whereas the fitting quality of *Pbcm* model remains little changed. Thus, both fitted *r*-regions at 500 K are better described by *Pbcm* structure. In particular, *Pbcm* space group describes the high *r*-region structure of both AN and ANL4 better than the low *r*-region, highlighting the reliability of *Pbcm* model to fit the average structure of AgNbO_3_-based antiferroelectrics. The overall inspection of the *R*_*w*_ values indicates that the local distortion of AN is more suitable to be described by *Pmc2_1_* space group while with increasing temperature and La content, the structure evolves towards *Pbcm* structure.

**Figure 2:**
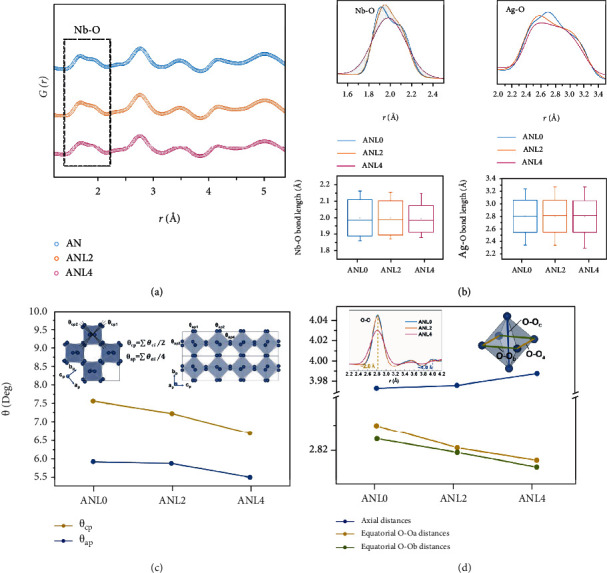
Local structural characteristics of AN, ANL2, and ANL4. (a) Experimental *G*(*r*) of AN, ANL2, and ANL4 with Nb-O pairs highlighted. (b) Partial Nb-O (upper left) and Ag-O (upper right) PDFs as obtained from PDF refinements. Box plots of Nb-O (bottom left) and Ag-O (bottom right) bond length distribution. The edges of the boxes indicate the standard deviations. (c) Oxygen octahedral tilting angles as a function of composition. The insets are schematic illustrations of AgNbO_3_ lattice viewed along *c*_*p*_ and *a*_*p*_ axes with the tilting (rotation) angles highlighted. (d) Equatorial O-O_a_ and O-O_b_ distances and axial O-O_c_ distances as a function of composition. The inset shows the partial O-O PDFs and the [NbO_6_] octahedron with specific O-O distances marked.

To shed light on the effect of La dopant on the local and intermediate structure of AgNbO_3_, the low *r*-region PDFs for AN, ANL2, and ANL4 are compared, as shown in Figure 2(a). Small but yet systematic differences are observed for the Nb-O pairs. The shoulder peak is gradually smeared from AN to ANL4, suggesting a more uniform distribution of Nb-O bond lengths. Partial Nb-O PDFs for different compositions are shown in Figure 2(b), and the Nb-O bond length distribution summarized from the calculated model is plotted below. Appreciable narrower spread of the Nb-O distances with increasing La content is observed, implying the suppressed off-center displacement of Nb^5+^. The Ag-O bond lengths, by contrast, show a more distorted coordination with the length values ranging between 2.3 Å and 3.2 Å. The highly distorted Ag^+^ displacement is primarily associated with its interstitial position, suggesting that Ag^+^ cations are more flexible than Nb^5+^ to accommodate the ionic size mismatch.

Apart from cation displacements, oxygen octahedral tilting is another significant structural characteristic. The calculated oxygen octahedral tilting angles of AN and their composition dependence are shown in Figure 2(c), along with schematics for the designation of tilting angles with respect to the pseudo perovskite *c*_*p*_ and *a*_*p*_ axis. These two types of tilting angles decrease with increasing La content, suggesting a suppressed octahedral tilting scheme. The decreased magnitude of tilting angles indicates a structural relaxation which occurs primarily to mitigate the undercoordination of oxygen. Besides, given that the change of octahedral tilting will inevitably alter the interatomic forces between the neighbouring cations, one can expect a notable correlation between the decreased octahedral tilting angle and suppressed cation displacement.

Due to the high sensitivity of neutron scattering to oxygen, the O^2-^ positions can be reliably characterized, facilitating analyses of the evolution of individual oxygen octahedron upon doping. Two types of O-O distances, that is, the distances in the (001) plane and the distances along the <001> direction are focused. The equatorial and axial O-O distances are around 2.8 Å and 4 Å, respectively, as highlighted by the partial O-O PDFs in Figure 2(d) Inset. The equatorial O-O distances are divided into two groups labelled O-O_a_ and O-O_b_. Interestingly, the equatorial O-O distances show a progressive contraction while the axial O-O distances follow an opposite trend, exhibiting an anisotropic variation upon La doping. The extended axial O-O distances, combined with the suppressed tilting along *c*_*p*_ axis, account for the experimental observation that the lattice parameter *c*_*p*_ shows progressive increase with increasing La content (Figure S3). Interestingly, the anisotropic change of the shape of the oxygen octahedron is consistently observed upon the change of temperature [10]. This suggests that, to a certain extent, La doping and elevated temperature modify the shape of individual oxygen octahedron in parallel ways.

### 2.2. Cation Displacement

To comprehensively understand the spatial variations of the atomic structure, RMC protocols are implemented to model the large length-scale atomic configurations. First, we compared the fitting result with the initial configuration of *Pbcm* and *Pmc2_1_* models. Though both models yield satisfactory results, the computation with the *Pbcm* model has reached a lower loss in both *G*(*r*) and *S*(*Q*) fitting, and the results are more consistent with the experimental data, compared with those using the *Pmc2_1_* model (see Figure S4). We assume that the higher symmetry of *Pbcm* space group makes it easier for the algorithm to find the initial moves during the early stage of fitting. Additionally, the subtle difference in ionic displacements between the two models makes no discernible influence (as has been confirmed) after random atom moves occur in RMC fitting process. Thus, all the RMC simulation results reported here are from the *Pbcm* initial model. The Bragg profiles and neutron total scattering data in both real and reciprocal spaces were fitted simultaneously, and the refined configuration provided a satisfactory fit to all datasets (Figure S5). AN and ANL4 are selected hereafter for comparison to highlight the effect of La dopant on the structural evolution.

**Figure 3:**
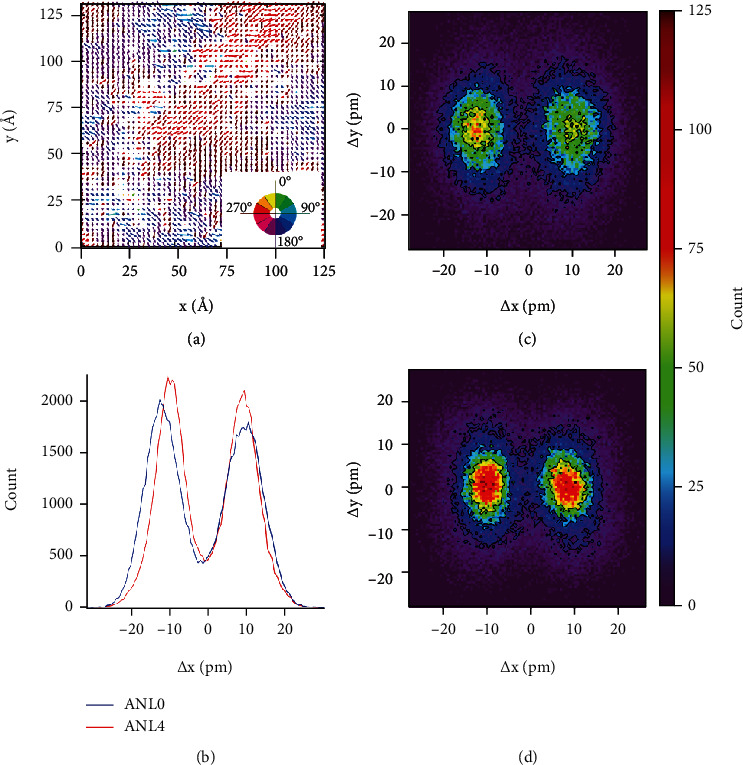
A complete view of the displacement of Nb atom projected onto the (110)_c_ plane (in-plane displacement). (a) Illustration of the Nb displacement; the arrows point the displacement direction, and the length represents the magnitude of the displacement. (b) Comparison of profiles of Nb in-plane displacement for AN and ANL4. (c, d) Illustration of the projection of Nb displacements of AN and ANL4, respectively.

For a clear demonstration, the spatial displacement of cations is decomposed into an in-plane component (displacement within the *ab* plane) plus an out-of-plane one (displacement along the *c* axis). The map of the in-plane displacement of Nb^5+^ for AN is shown in Figure 3(a), where several regions of correlated orientation can be seen. Figure 3(b) compares the profile of the displacement distribution along the horizontal direction for AN and ANL4, where a marginally decreased off-center displacement is observed for ANL4. Figures 3(c) and 3(d) depict the distribution of Nb^5+^ displacement for AN, which exhibits an obvious two-site preferred distribution along the <110>_c_ direction, mirroring their antiparallel displacement. The magnitude of the antipolar displacement is around 10 pm, comparable to the experimental observations by scanning transmission electron microscopy [26]. For ANL4, the antipolar pattern is mostly retained but with a narrowed spread (Figure 3(d)). This phenomenon is generally consistent with the suppressed distortion of the Nb-O bond, as suggested above by the small-box PDF refinement.

The distribution of Ag appears to be more complicated because there are two distinct sites for Ag, i.e., Ag1 and Ag2. Figures S6(a) and S6(b) present the maps of the Ag1 and Ag2 displacement for AN. The distribution of Ag1 follows a bimodal scenario while the distribution of Ag2 appears to be unimodal, which is consistent with the *Pbcm* symmetry. As for ANL4, the displacement of Ag is arranged in a similar way to that of AN while its distribution becomes narrower (Figures S6(c) and S6(d)). This contrast is further ascertained by the sharper peaks for ANL4 in corresponding profiles (Figure S6(e) and S6(f)). The above observations agree with the known trends that La doping tends to suppress the cation displacement in AgNbO_3_, primarily due to its less covalent bonding with oxygen [5].

**Figure 4:**
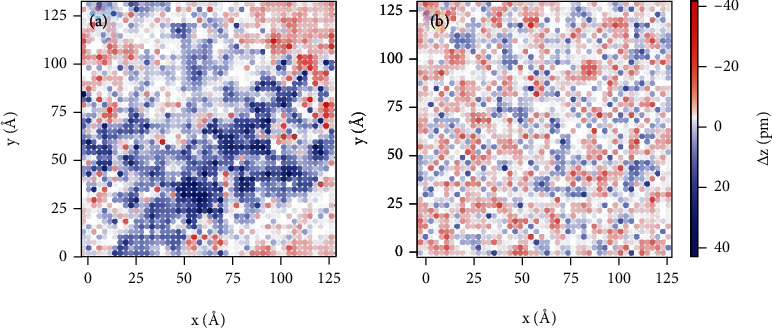
Projection of the Nb <001>_c_ displacement. <001>_c_ displacement for (a) AN and (b) ANL4. The color-scale bar reflects the magnitude of the displacement. The horizontal and vertical axes correspond to the <100> and <010> directions, respectively.

Next, we extract the <001>_c_ displacement of Nb^5+^ for analysis, and the configuration is visualized in Figure 4. Interestingly, the distribution of the <001>_c_ displacement of Nb cations in AN is not completely random but rather displays certain degree of aggregation, featuring correlated regions several nanometers in size, inside which the cation displacements are along the same direction, as highlighted by the color contrast in Figure 4(a). Within these nanoregions, the maximum displacement of Nb^5+^ ions is about 30 pm, and the clusters are around 6-10 nm in size. Nanoregions are observed for the <001>_c_ displacement of Ag cations as well with a notable coupling with that of Nb cations (Figure S7). As expected, Ag cations exhibit larger displacements than Nb within the same regions, conforming to the larger distortion of Ag-O as suggested by the PDF refinement.

In contrast to the correlated nanoregions in pure AN, the distribution of the <001>_c_ displacement of Nb cations appears to be more random and fragmental for ANL4 (Figure 4(b)), implying that the incorporation of La disrupts the short-range order of the cation displacements. In addition to the weaker correlation, the maximum magnitude of the displacements also decreases to 20 pm. Inspection of the configuration for Ag cations demonstrates a similar disordered pattern in ANL4 (Figure S7(b)). For more details, the supercell with a height of 12 nm is sliced into 32 layers along the *z*-direction (each 3.75 Å thick, close to the scale of one unit cell), and the projection of the <001>_c_ displacement of each layer is presented in Figure S8. Evidently, the coupled clusters of the <001>_c_ displacement have been broken in ANL4. With the disappearance of correlated clusters, the distribution of the displacement also becomes smaller in magnitude. These observations suggest that La doping alters the cation displacement of AgNbO_3_ in both magnitude and distribution.

### 2.3. A-Site Vacancy Ordering

**Figure 5:**
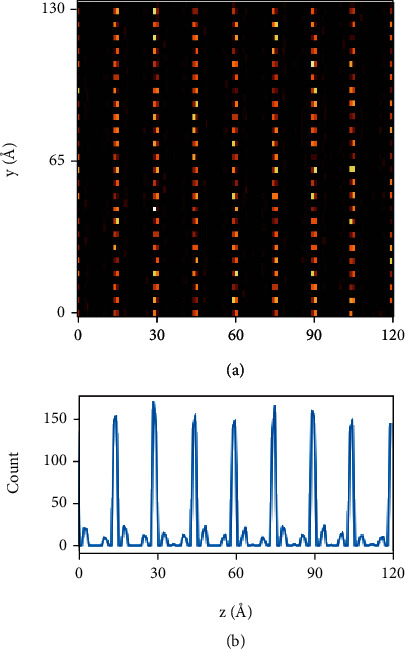
An illustration of ordered A-site vacancies in ANL4. The *y* and *z* axes correspond to the <010> and <001> directions, respectively. (a) An in-plane detailed view of vacancy positions. (b) Histogram of vacancies on different layers.

In Ag_1−3x_La_x_NbO_3_, for each La that substitutes for Ag, two A-site vacancies will be induced to maintain charge neutrality. Generally, at sufficiently high concertation, vacancies are prone to aggregate in order to minimize the free energy of the system [27, 28]. The configuration of A-site vacancies in ANL4 is explored by analyzing the supercell reconstructed by the RMC method. As shown in Figure 5(a), the A-site vacancies of ANL4 are preferentially distributed in every twice [001]_c_ row to produce alternative layers with vacant and occupied A-site positions. Figure 5(b) displays the corresponding histogram for the distribution of A-site vacancies. Upon close examination, the vacancies show an obvious preference for the Ag2 position whereas the probability of its presence at the Ag1 position is low. This suggests that the nonequivalent coordination environment of A-site cations could be the origin of the distinct array of vacancies. The ordering of Ag vacancies is reminiscent of analogous A-site-deficient perovskites in which the A-site vacancies serve as mediators to relieve bond strain of the lattice [29, 30]. Layered ordering of the A-site cations is commonly observed in cation-deficient perovskites such as La_1/3_NbO_3_ and La_2/3_TiO_3_ [31, 32]. These A-site-deficient compounds usually contain modulated structures with alternating (001) layers of fully and partly occupied A-sites. Second-order Jahn-Teller effect, where highly charged d^0^ cations on the B-site moves out of the center of their coordination polyhedral, is an important mechanism for stabilizing A-site cation ordering and relieving the bonding instability caused by ordering [33, 34]. Therefore, it is likely that for La-doped AgNbO_3_, the specific ordering of A-site vacancies and variation in oxygen octahedral tilting angle generates a cooperative effect to stabilize the structure. It should be noted that the analysis of vacancy ordering based on RMC describes the models of reasonable probability, while multiple competing vacancy configurations are likely to coexist due to the abundant defects in polycrystalline ceramics and thermal fluctuations on ambient conditions [35]. More deterministic information might be obtained if the energy difference is enlarged when the concentration of vacancies is high enough to populate a significantly distinct configuration.

### 2.4. Dielectric Properties

The disrupted nanoscale correlations upon La doping appear to induce a gradual change in the dielectric properties. Figure S9 shows the temperature-dependent dielectric constant of AN, ANL2, and ANL4 measured on heating and cooling, respectively. An anomaly that is called freezing temperature [36, 37] *T*_*f*_ is observed for AN at ~175°C, which becomes greatly smeared for ANL2 and ANL4. Generally, *T*_*f*_ indicates the hopping dynamics of Nb^5+^ among the eight ⟨111⟩_c_ positions in the lattice. Below *T*_*f*_, the two sites along the <110>_c_ direction are preferentially occupied thus initiating the antiparallel order of Nb^5+^ displacements [38]. This mechanism implies that *T*_*f*_ is closely associated with cation displacements, especially the <001>_c_ displacement correlations. It is thus reasonable to assume that the disrupted nanoscale correlations are the primary origin of the smeared *T*_*f*_ of ANL2 and ANL4. Considering the fact that the eight-site model of cation displacement is commonly observed in other perovskite compounds such as NaNbO_3_ [30] and PbMg_1/3_Nb_2/3_O_3_ [17], the scenario proposed here may convincingly account for the displacive disorder in these relevant perovskites and corresponding variation in dielectric properties.

## 3. Conclusion

In summary, small-box refinement and large-box simulation have been incorporated to examine the structural characteristics of pure and La-doped AgNbO_3_ at different length scales. Modelling of the PDF reveals that the noncentrosymmetric space group *Pmc2_1_* provides a better description of the room-temperature structure of AgNbO_3_. The incorporation of La tends to suppress the structural distortion with respect to bond length distributions as well as octahedral tilting. The large atomic configuration established by the RMC method demonstrates that the antipolar displacement of cations within the (001)_c_ plane are suppressed upon La doping. The displacements of Nb and Ag along the <001>_c_ direction are qualitatively coherent in pure AgNbO_3_, and the alignment of these clusters gives rise to correlated nanoregions several nanometers in size, which appears to be disrupted in La-doped AgNbO_3_. The disordered nanoscale correlations of cations suppress the anomaly of dielectric constant around the freezing temperature. A-site vacancies induced by the aliovalent dopants are preferentially displaced at the Ag2 site and tend to order into alternate (001) layers. Our work has revealed that local structural characteristics and their assembly into the long-range average configurations are essential to depict a clear picture of the atomic structure of AgNbO_3_. These insights provide guidelines for future exploration of the structural characteristics of perovskite-based antiferroelectrics as well as the underlying structure-property correlation.

## 4. Materials and Methods

### 4.1. Synthesis and Measurement of Dielectric Properties

Ceramic samples of pure and La-doped AgNbO_3_ (Ag_1-3x_La_x_NbO_3_) were prepared by solid-state reaction according to the synthesis route described in references [16, 39]. Here, three compositions, AgNbO_3_, Ag_0.94_La_0.02_NbO_3_, and Ag_0.88_La_0.04_NbO_3_ (abbreviated as AN(ANL0), ANL2, and ANL4, respectively), were specifically chosen for detailed structural characterization.

For the measurement of dielectric properties, the samples were coated with Ag electrodes, and the measurement was conducted in a furnace using an LCR meter (TH2828S; Changzhou Tonghui).

### 4.2. Total Scattering Measurements

Neutron total scattering data were collected at both 300 K and 500 K at Spallation Neutron Source (SNS)'S NOMAD beamline of Oak Ridge National Laboratory. The ceramic pellets of different compositions were crushed into powders, followed by annealing at 500°C for 3 h in order to remove possible residual stresses, and the powders were then sealed in quartz capillaries for measurement. The total scattering data cutoff at a maximum wavevector transfer *Q*_max_ of 40 Å^−1^ were used for Fourier transform to the reduced pair distribution function. Small-box modelling of the PDF data was conducted with PDFgui packages [40]. The samples employed in this work were processed from the same batch as in ref. [16], where the phase purity was confirmed by neutron diffraction. In the unit cell of ANL2 and ANL4 composition, 2 mol% or 4 mol% La atoms were put at the A-site with no preference for the distinct crystallographic Ag sites.

### 4.3. Structure Refinements

Large-box modelling was performed by Reverse Monte Carlo (RMC) simulations with the RMCprofile software [41] using a 24 × 24 × 8 supercell (~13.3 × 13.4 × 12.5 nm^3^) containing 184320 atoms. Specifically, the model for AN contains 36864 Ag, 36864 Nb, and 110592 O atoms. For ANL4, 32558 Ag, 36864 Nb, 110592 O, 1475 La, and 2831 V_a_ (vacancies) were included in the model. La atoms and vacancies were introduced by randomly changing 4% of Ag atoms to La atoms and 8% of Ag atoms to vacancies at the same time. Positions of La atoms, Ag atoms, and vacancies are allowed to swap with 20% probability. Various simulation durations were adopted according to certain parameter settings and corresponding convergence rates. About 15 million moves were generated (~1 million accepted) per the algorithm to simultaneously fit the Bragg profiles and neutron total scattering data in both real and reciprocal spaces. For most simulation parameters, the default settings are adopted. We use atom distance (defined as the distance window in RMCprofile) to constrain atom moves. During the simulations, weights between Bragg profiles and neutron total scattering data were adjusted dynamically according to their fitting condition.

Additional RMC simulations were performed to verify the reliability of the fitting result. The fitting was separated to two stage. In the first stage, the atom moving range was set at 0.8 Å. The cutoff boundary was decided correspond to *G*(*r*) profiles. An automated optimization of weights among the *G*(*r*), *S*(*Q*), and Bragg profiles was enabled. The program was allowed to accept some moves that would make the loss rise slightly. In the second stage, the atom moves were limited to 0.2 Å, and the optimization process was cancelled. The evolution of acceptance rate and fitting loss was recorded to evaluate the influence of stage 1. Similar conclusions of atom displacement and vacancy arrangement could be drawn, and the validity of the RMC model is solid. See more details in Figure S11 and S12 and discussions therein.

## Data Availability

The data used to support the findings of this study are available from the corresponding authors upon request.

## Supplementary Materials

Supplementary Materials Figure S1: PDFs of AN (500 K). Figure S2: PDF refinements for ANL4. Figure S3: Lattice parameter *c*_*p*_ as a function of La content. Figure S4: a comparison of the fitting quality between calculations with starting model of Pbcm and Pmc21 space groups. Figure S5: results of data fitting for AN and ANL4 at 300 K. Figure S6: illustration of the projection of Ag displacements. Figure S7: projection of the <001>_c_ displacement of Ag. Figure S8: overall view of the <001>_c_ Nb atom displacement. Figure S9: overall view of the <001>_c_ Ag atom displacement. Figure S10: temperature-dependent dielectric constant of AN, ANL2, and ANL4. Figure S11: RMC fitting results for the verification test. Figure S12: analysis for the RMC verification test.
