# Supplementary material for: Local Atomic Configuration in Pristine and A-Site Doped Silver Niobate Perovskite Antiferroelectrics
Source: Research (Wash D C). 2022 Feb 25;2022:9782343. doi: 10.34133/2022/9782343 (PMC8898335; doi:10.34133/2022/9782343)
Supplement: Supplementary Materials — Figure S1: PDFs of AN (500 K). Figure S2: PDF refinements for ANL4. Figure S3: Lattice parameter cp as a function of La content. Figure S4: a comparison of the fitting quality between calculations with starting model of Pbcm and Pmc21 space groups. Figure S5: results of data fitting for AN and ANL4 at 300 K. Figure S6: illustration of the projection of Ag displacements. Figure S7: projection of the <001>c displacement of Ag. Figure S8: overall view of the <001>c Nb atom displacement. Figure S9: overall view of the <001>c Ag atom displacement. Figure S10: temperature-dependent dielectric constant of AN, ANL2, and ANL4. Figure S11: RMC fitting results for the verification test. Figure S12: analysis for the RMC verification test. [file 9782343.f1.zip › Research SI 220107 resubmisson.docx]

***Supporting Information for:***

**Local Atomic Configuration in Pristine and A-site Doped Silver Niobate Perovskite Antiferroelectrics**

Jing Gao,^1, †^ Wei Li,^1, †^ Jue Liu,^2^ Qian Li,^1,*^ and Jing-Feng Li^1,*^

*^1^ State Key Laboratory of New Ceramics and Fine Processing, School of Materials Science and Engineering, Tsinghua University, Beijing, 100084, China*

*^2^ Neutron Scattering Division, Oak Ridge National Laboratory, Oak Ridge, Tennessee, 37831, USA*

^†^ These authors contributed equally to this work: Jing Gao, Wei Li

^*^ Correspondence should be addressed to Jing-Feng Li; jingfeng@mail.tsinghua.edu.cn and Qian Li; qianli_mse@tsinghua.edu.cn


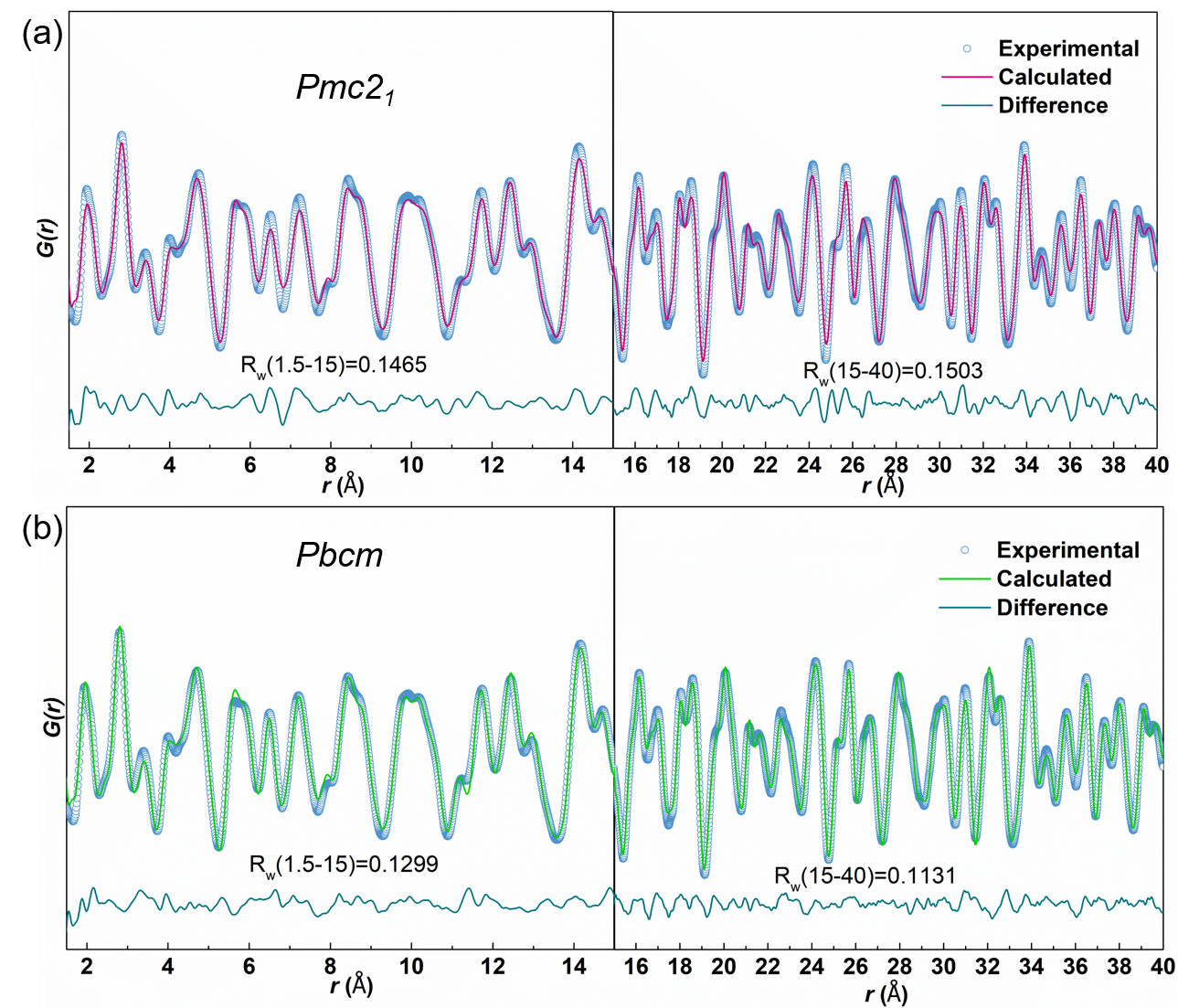


**Figure S1.** **PDFs of AN (500 K).** Experimental patterns fitted with *Pmc2_1_* (a) and *Pbcm* (b) space groups for the r=1.5-15 Å and the 1-40 Å ranges.


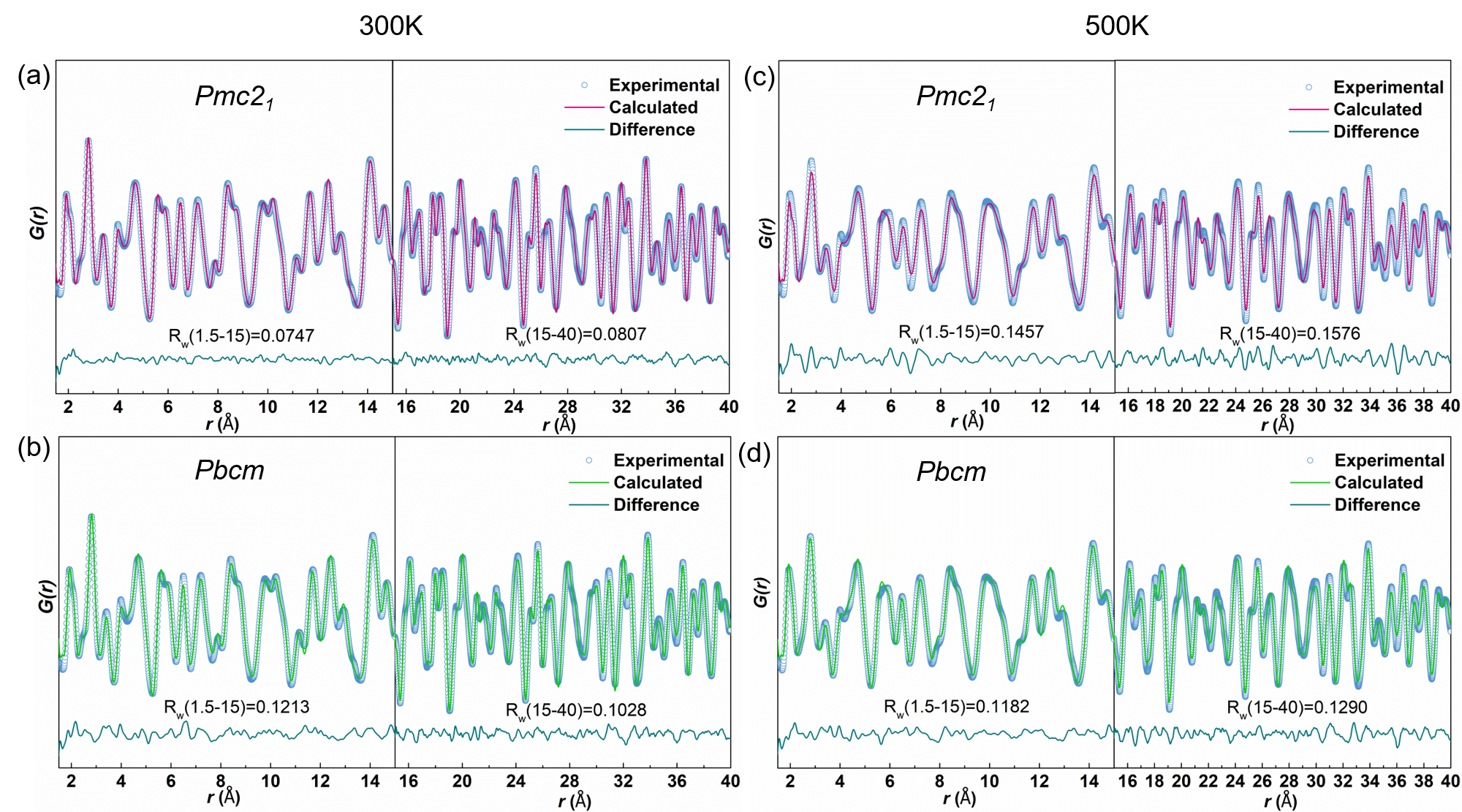


**Figure S2.** **PDF refinements for ANL4.** Data collected at 300 K (a, b) and 500 K (c, d).





**Figure S3.** **Lattice parameter *c_c_* as a function of La content.**


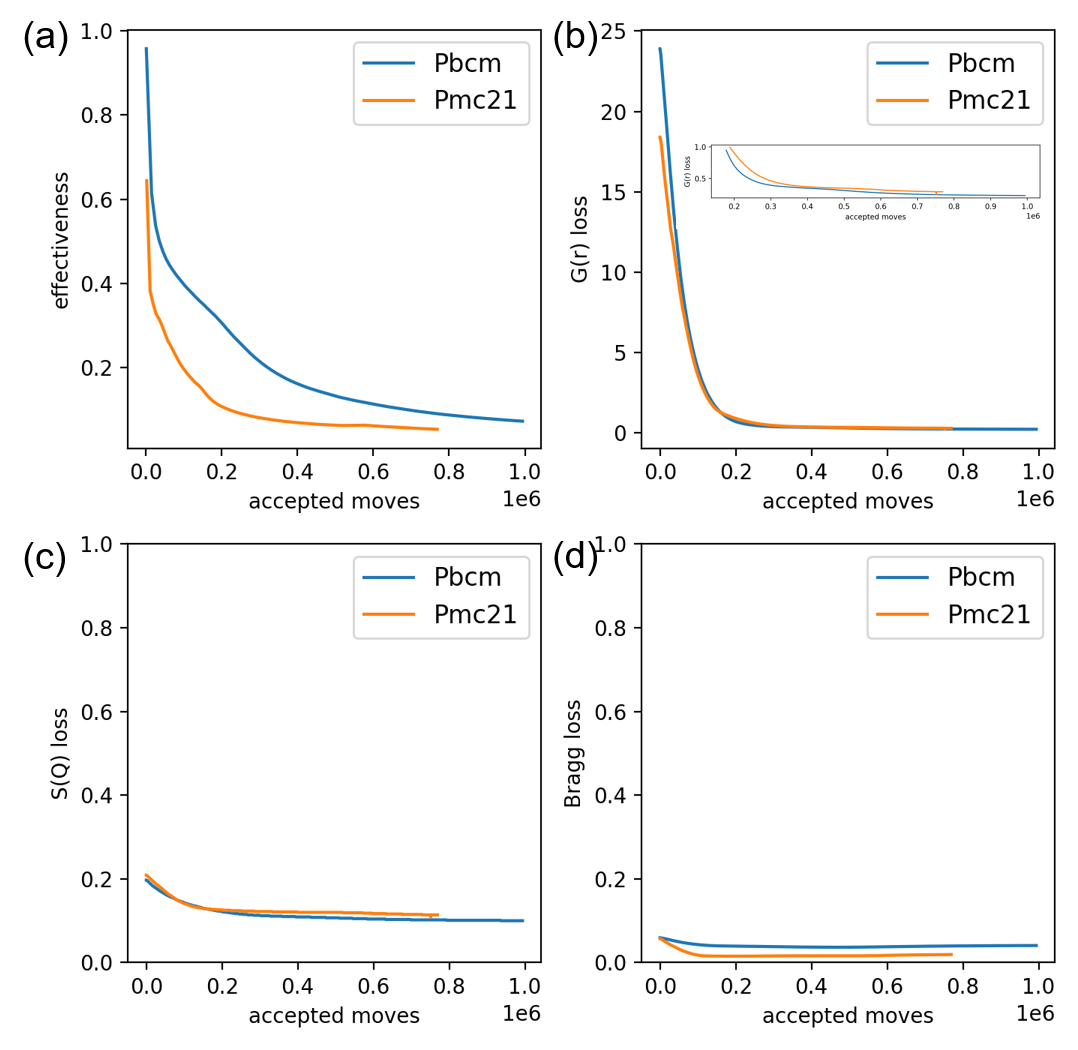


**Figure S4.** **A comparison of the fitting quality between calculations with starting model of *Pbcm* and *Pmc2_1_* space groups.** Though the fitting with *Pmc2_1_* space group yields lower loss in Bragg fitting, it causes higher loss in *G(r)* and *S(Q)* fitting (which shows higher sensitivity to local structure). Overall, higher fitting effectiveness has been achieved by *Pbcm* model.


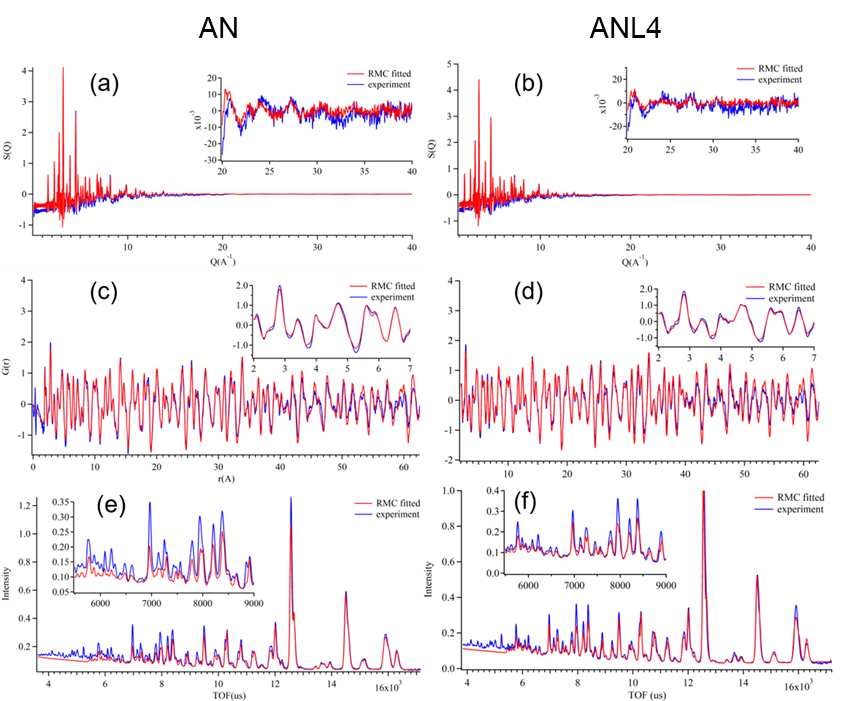


**Figure S5.** **Results of data fitting for AN and ANL4 at 300K.** Red and blue lines represent experimental and calculated signals. (a, c, e) Fitting results for AN. (b, d, f) Fitting results for ANL4. (a, b) Neutron total scattering function S(Q), (c, d) Fourier transform of total scattering G(r), (e, f) Bragg profile.


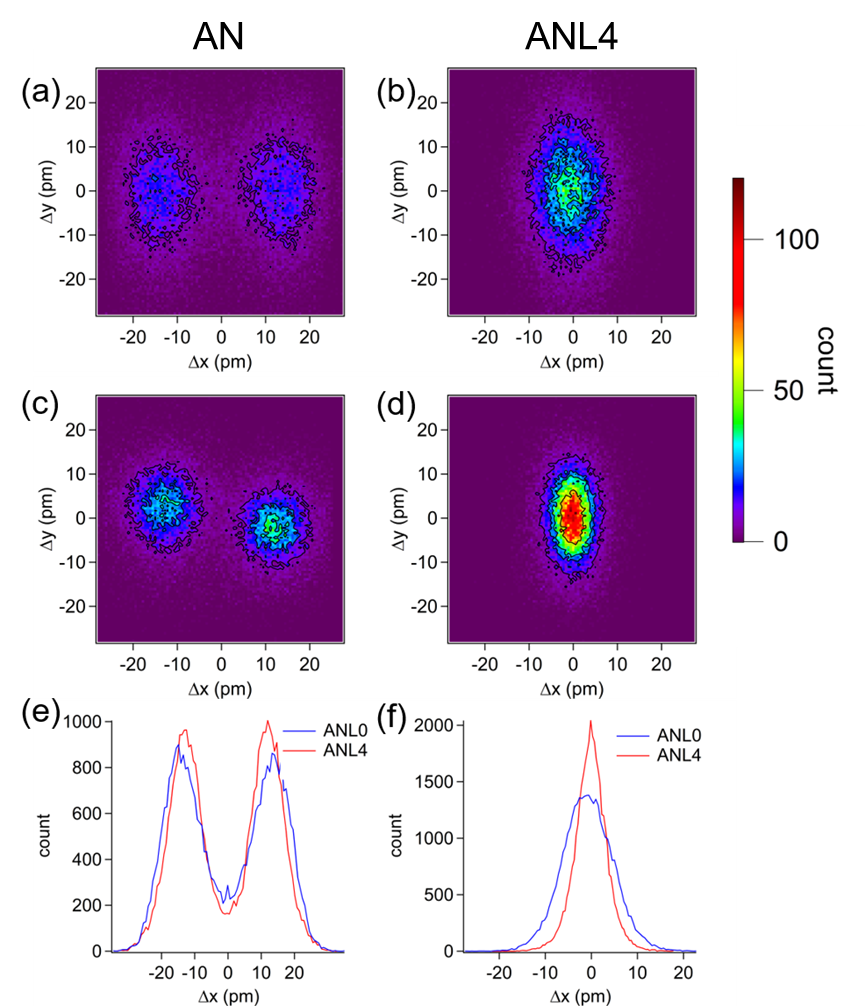


**Figure S6. Illustration of the projection of Ag displacements.** (a - d**)** Displacements of Ag1 and Ag2 for AN and ANL4, respectively. (e, f) Corresponding profiles of the displacement.


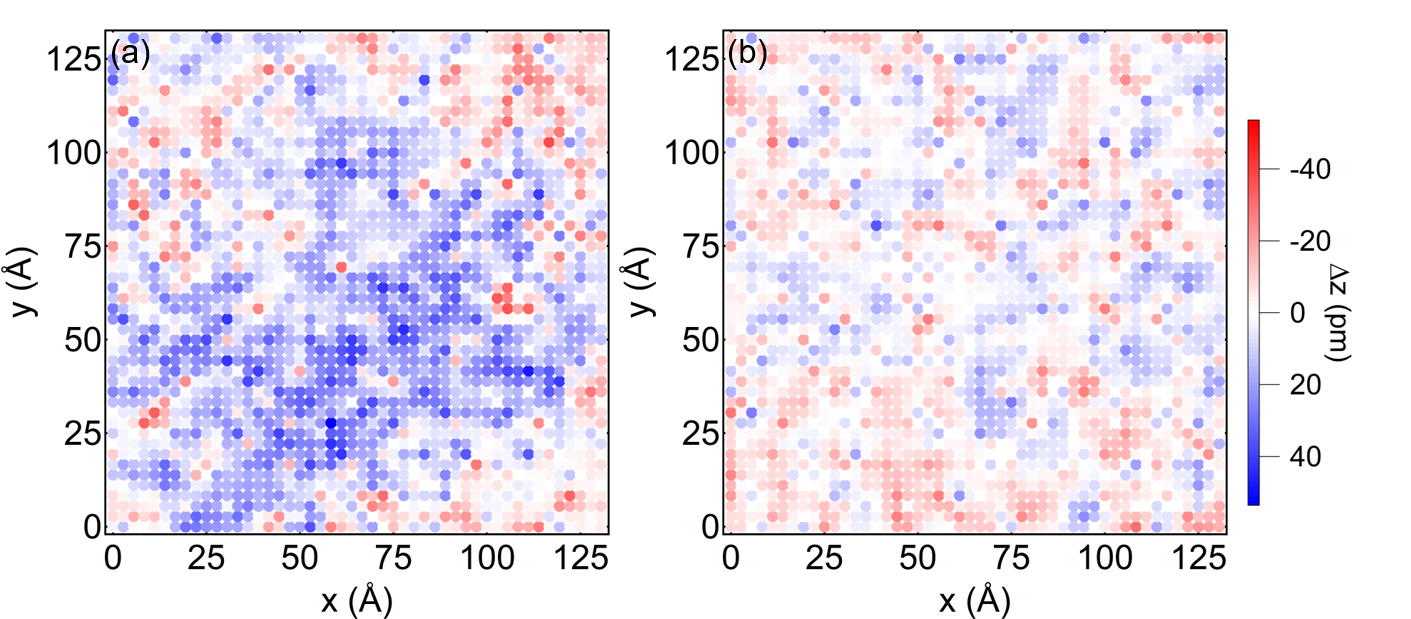


**Figure S7.** **Projection of the <001>_c_ displacement of Ag.** The distribution of the <001>_c_ displacement for (a) AN and (b) ANL4. The color-scale bar reflects the magnitude of the displacement. The horizontal and vertical axes correspond to the <100> and <010> directions, respectively.


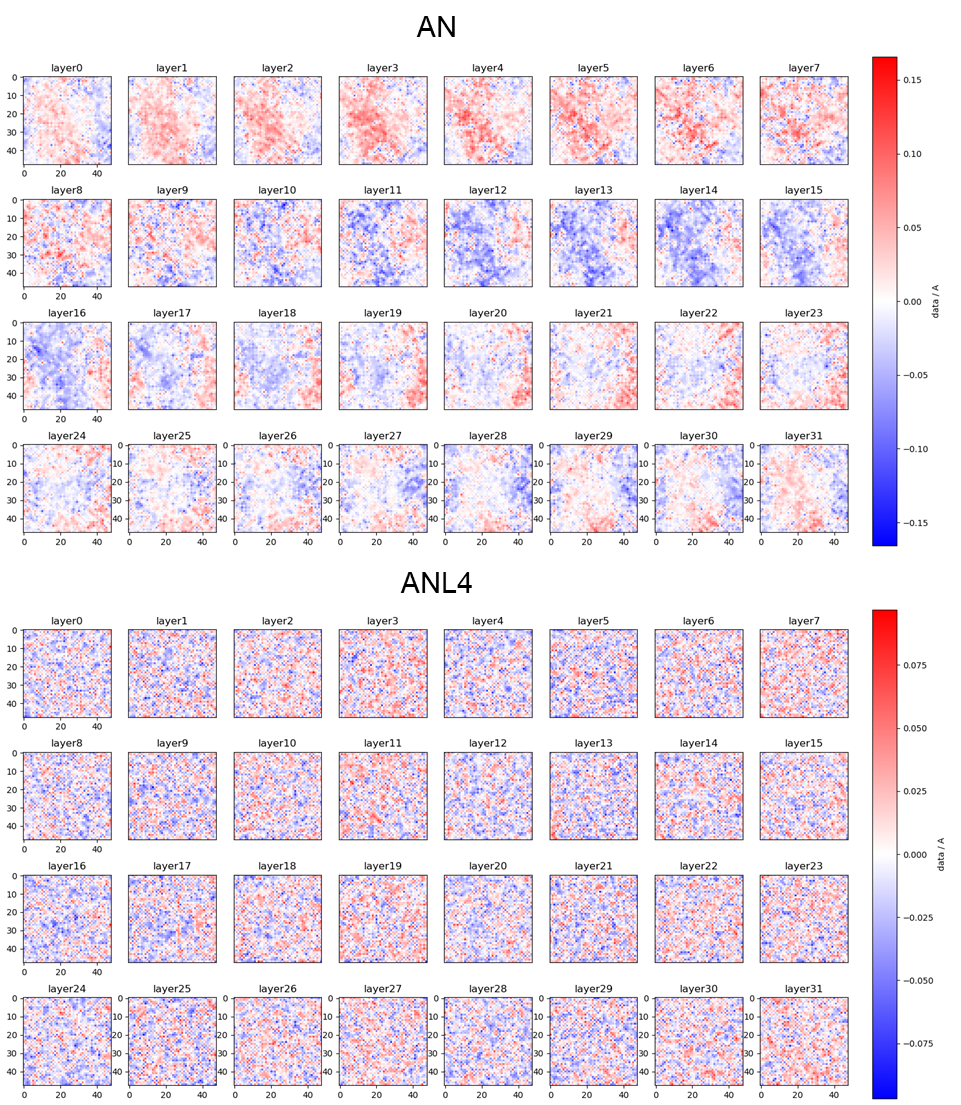


**Figure S8.** **Overall view of the <001>_c_ Nb atom displacement.** Projection of <001>_c_ Nb atom displacement for AN (a, b) and ANL4 (c, d) sliced along the [001]_c_ direction (z-axis).


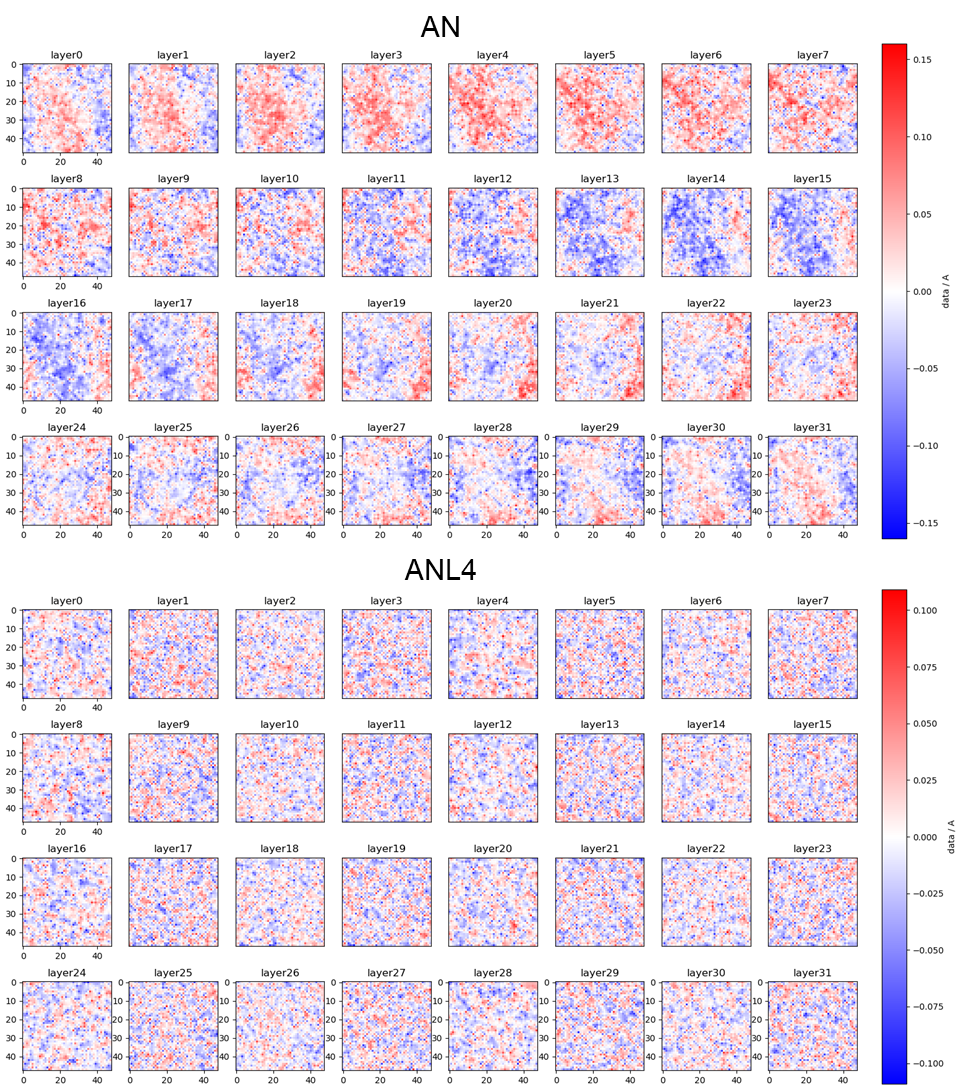


**Figure S9.** **Overall view of the <001>_c_ Ag atom displacement.** Projection of <001>_c_ Ag atom displacement for AN (a, b) and ANL4 (c, d) sliced along the [001]_c_ direction (z-axis).


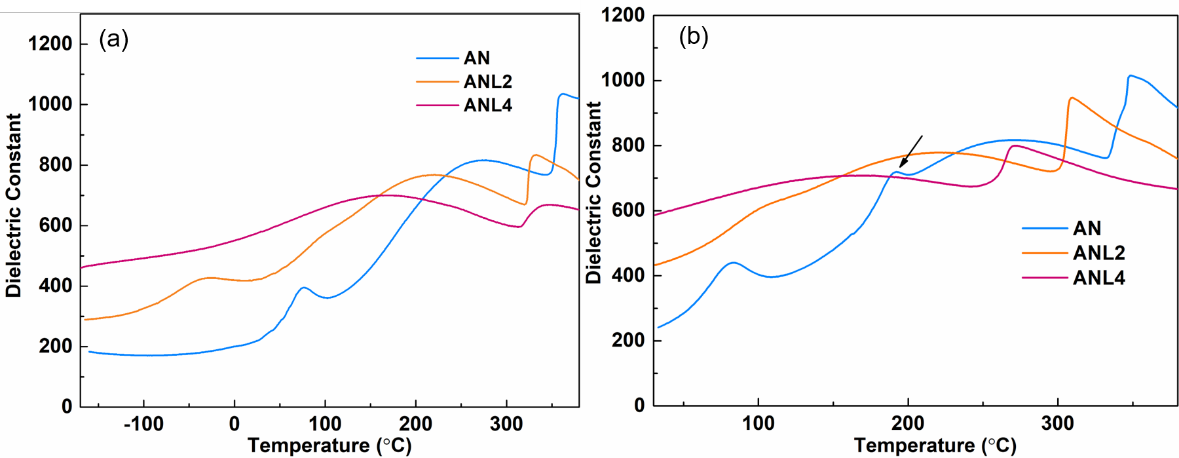


**Figure S10.** **Temperature-dependent dielectric constant of AN, ANL2 and ANL4.** (a, b) Data collected on heating and cooling, respectively. The arrow in (b) indicates the freezing temperature T_f_.


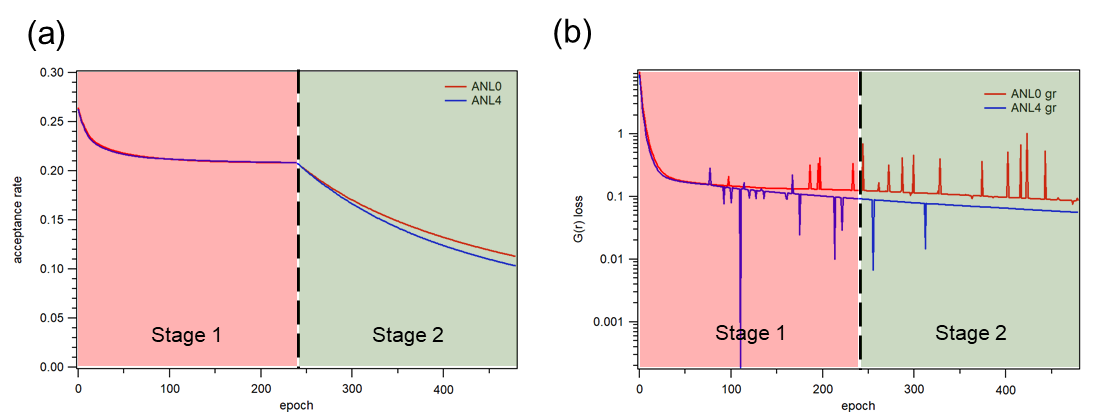


**Figure S11. RMC fitting results for the verification test.** (a) Atom moves acceptance rate related with fitting epochs. (b) G(r) loss decreases consistently during the fitting process.

**
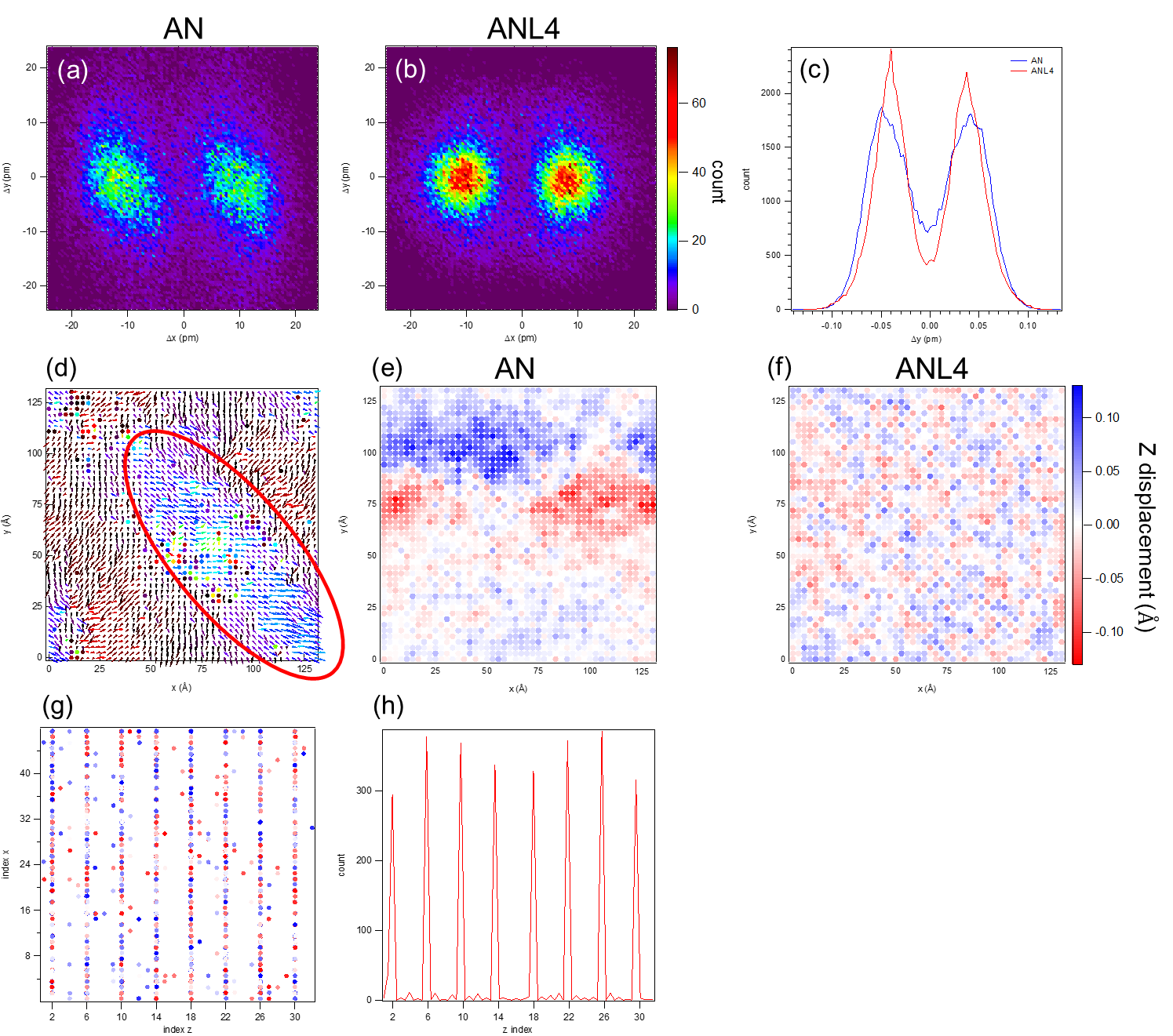
**

**Figure S12. Analysis for the RMC verification test.** (a, b) in-plane Nb displacement for AN and ANL4 (c) slice view along x axis (d) arrow plot of in-plane Nb displacement (e, f) out-of-plane Nb displacement for AN and ANL4 (g) vacancy distribution in x-z plane (f) histogram of vacancies along z axis.

RMC modeling uses a loss function to determine whether atom moves should be accepted. To verify if the fitting reached its global minimum instead of local minimum and to guarantee sufficient quantity of total moves, another simulation was performed for comparison **(Figure S11**). Here, one epoch refers to one check point during the simulation, which represents ~100,000 moves generated. The first 240 epochs applied a set of loose fitting parameters to allow the program to jump out of potential local minimums (stage 1). And the following 240 epochs were set the same as other simulations so as to converge the result to its final state (stage 2). A turning point occurred at the dividing epoch in **Figure S11(a)**, which is directly related with the strict limitation of the following fitting process. It is clear that with less strict fitting parameters the acceptance rate was significantly improved in stage 1. The drop of acceptance rate after the changing of fitting parameters proves that most of the accepted moves were generated as trial moves which were forbidden in stage 2.

**Figure S11(b)** gives an inspection of the fitting loss and shows that the loss decreased rapidly from the initial configuration to about epoch 20, and then slowly approached its minimum. No turning point can be observed around epoch 240, indicating that atom moves were accepted in similar conditions before and after the adjustment of simulation parameters. This is a strong evidence that the fitting was already converged to a small distance window after the early moves, consistent with the trend of acceptance rate.

Further analysis of the fitting result confirmed that with the new fitting method, the conclusions remained consistent with the former ones (**Figure S12**). With these results it is clear that the simulation reached its global minimum quickly and only performed small atom moves afterwards. Therefore, the current fitting model is sufficient and the results are reliable.
