# Supplementary figures and images for: Local Atomic Configuration in Pristine and A-Site Doped Silver Niobate Perovskite Antiferroelectrics
Source: Research (Wash D C). 2022 Feb 25;2022:9782343. doi: 10.34133/2022/9782343 (PMC8898335; doi:10.34133/2022/9782343)

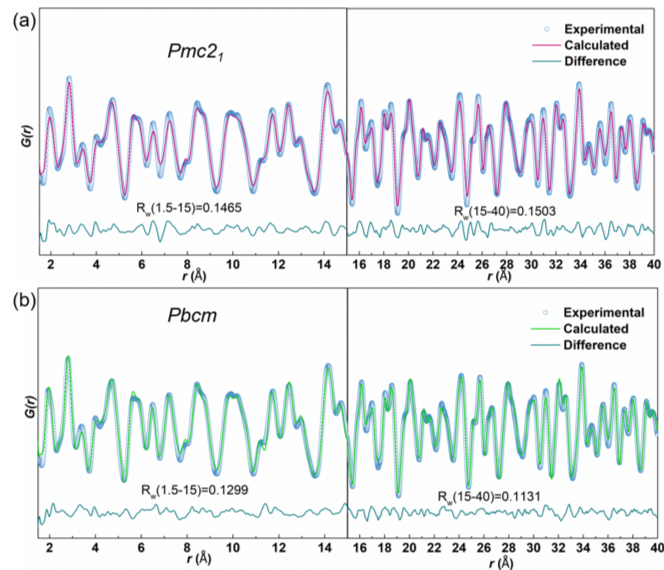

Supplement: Supplementary Materials — Figure S1: PDFs of AN (500 K). Figure S2: PDF refinements for ANL4. Figure S3: Lattice parameter cp as a function of La content. Figure S4: a comparison of the fitting quality between calculations with starting model of Pbcm and Pmc21 space groups. Figure S5: results of data fitting for AN and ANL4 at 300 K. Figure S6: illustration of the projection of Ag displacements. Figure S7: projection of the <001>c displacement of Ag. Figure S8: overall view of the <001>c Nb atom displacement. Figure S9: overall view of the <001>c Ag atom displacement. Figure S10: temperature-dependent dielectric constant of AN, ANL2, and ANL4. Figure S11: RMC fitting results for the verification test. Figure S12: analysis for the RMC verification test. [file 9782343.f1.zip › S1.png]

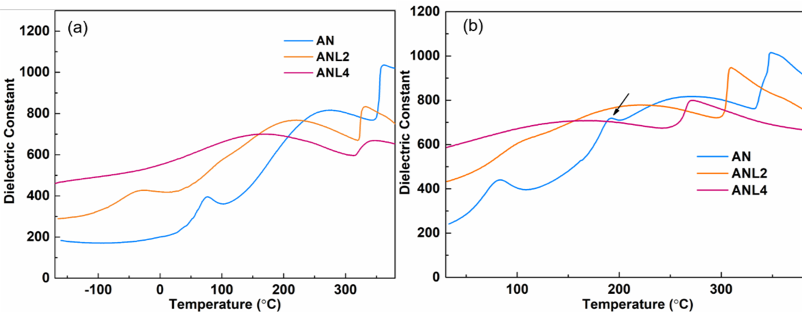

Supplement: Supplementary Materials — Figure S1: PDFs of AN (500 K). Figure S2: PDF refinements for ANL4. Figure S3: Lattice parameter cp as a function of La content. Figure S4: a comparison of the fitting quality between calculations with starting model of Pbcm and Pmc21 space groups. Figure S5: results of data fitting for AN and ANL4 at 300 K. Figure S6: illustration of the projection of Ag displacements. Figure S7: projection of the <001>c displacement of Ag. Figure S8: overall view of the <001>c Nb atom displacement. Figure S9: overall view of the <001>c Ag atom displacement. Figure S10: temperature-dependent dielectric constant of AN, ANL2, and ANL4. Figure S11: RMC fitting results for the verification test. Figure S12: analysis for the RMC verification test. [file 9782343.f1.zip › S10.png]

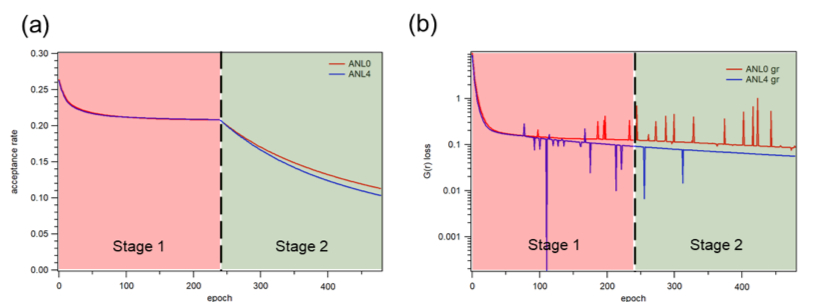

Supplement: Supplementary Materials — Figure S1: PDFs of AN (500 K). Figure S2: PDF refinements for ANL4. Figure S3: Lattice parameter cp as a function of La content. Figure S4: a comparison of the fitting quality between calculations with starting model of Pbcm and Pmc21 space groups. Figure S5: results of data fitting for AN and ANL4 at 300 K. Figure S6: illustration of the projection of Ag displacements. Figure S7: projection of the <001>c displacement of Ag. Figure S8: overall view of the <001>c Nb atom displacement. Figure S9: overall view of the <001>c Ag atom displacement. Figure S10: temperature-dependent dielectric constant of AN, ANL2, and ANL4. Figure S11: RMC fitting results for the verification test. Figure S12: analysis for the RMC verification test. [file 9782343.f1.zip › S11.png]

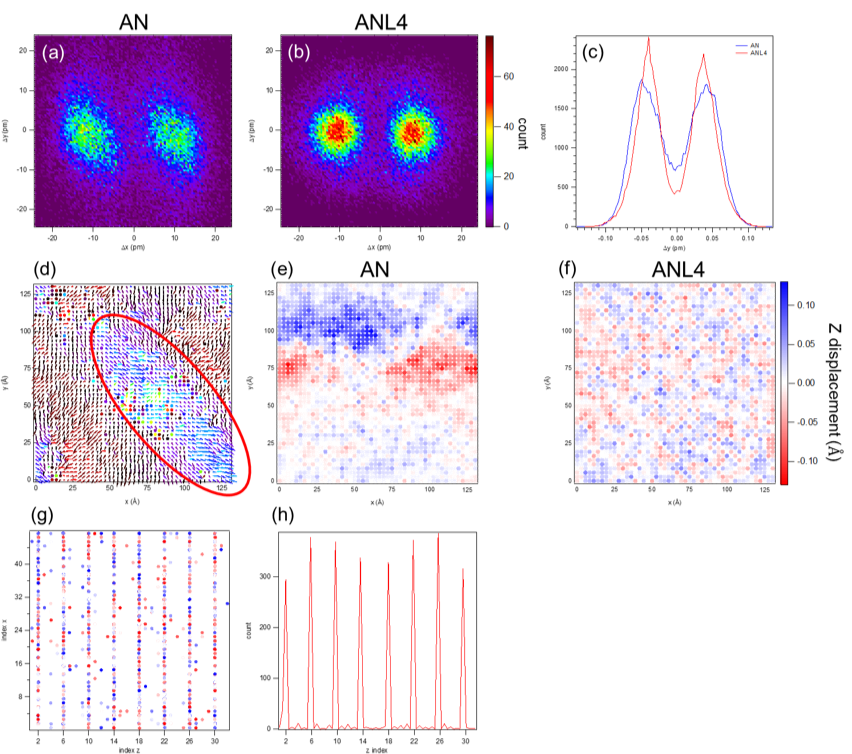

Supplement: Supplementary Materials — Figure S1: PDFs of AN (500 K). Figure S2: PDF refinements for ANL4. Figure S3: Lattice parameter cp as a function of La content. Figure S4: a comparison of the fitting quality between calculations with starting model of Pbcm and Pmc21 space groups. Figure S5: results of data fitting for AN and ANL4 at 300 K. Figure S6: illustration of the projection of Ag displacements. Figure S7: projection of the <001>c displacement of Ag. Figure S8: overall view of the <001>c Nb atom displacement. Figure S9: overall view of the <001>c Ag atom displacement. Figure S10: temperature-dependent dielectric constant of AN, ANL2, and ANL4. Figure S11: RMC fitting results for the verification test. Figure S12: analysis for the RMC verification test. [file 9782343.f1.zip › S12.png]

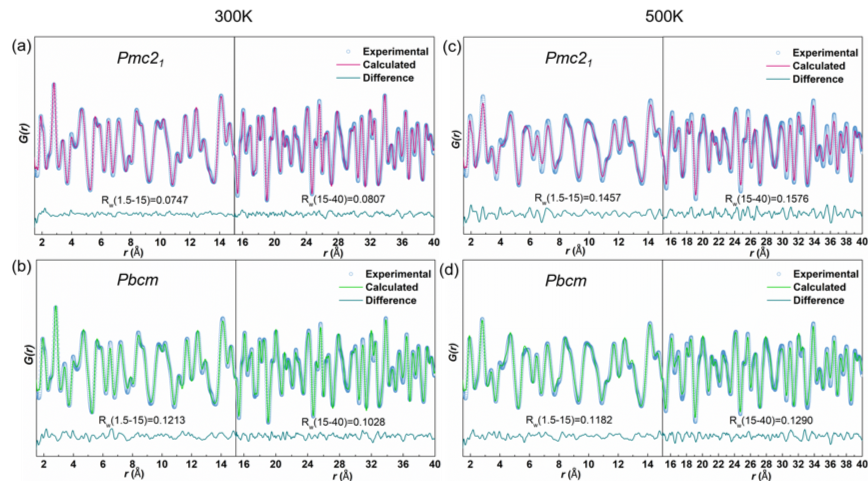

Supplement: Supplementary Materials — Figure S1: PDFs of AN (500 K). Figure S2: PDF refinements for ANL4. Figure S3: Lattice parameter cp as a function of La content. Figure S4: a comparison of the fitting quality between calculations with starting model of Pbcm and Pmc21 space groups. Figure S5: results of data fitting for AN and ANL4 at 300 K. Figure S6: illustration of the projection of Ag displacements. Figure S7: projection of the <001>c displacement of Ag. Figure S8: overall view of the <001>c Nb atom displacement. Figure S9: overall view of the <001>c Ag atom displacement. Figure S10: temperature-dependent dielectric constant of AN, ANL2, and ANL4. Figure S11: RMC fitting results for the verification test. Figure S12: analysis for the RMC verification test. [file 9782343.f1.zip › S2.png]

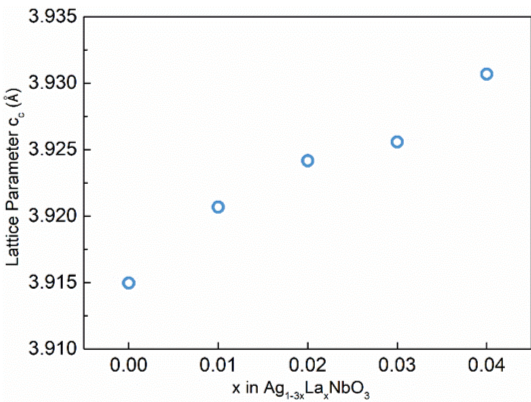

Supplement: Supplementary Materials — Figure S1: PDFs of AN (500 K). Figure S2: PDF refinements for ANL4. Figure S3: Lattice parameter cp as a function of La content. Figure S4: a comparison of the fitting quality between calculations with starting model of Pbcm and Pmc21 space groups. Figure S5: results of data fitting for AN and ANL4 at 300 K. Figure S6: illustration of the projection of Ag displacements. Figure S7: projection of the <001>c displacement of Ag. Figure S8: overall view of the <001>c Nb atom displacement. Figure S9: overall view of the <001>c Ag atom displacement. Figure S10: temperature-dependent dielectric constant of AN, ANL2, and ANL4. Figure S11: RMC fitting results for the verification test. Figure S12: analysis for the RMC verification test. [file 9782343.f1.zip › S3.png]

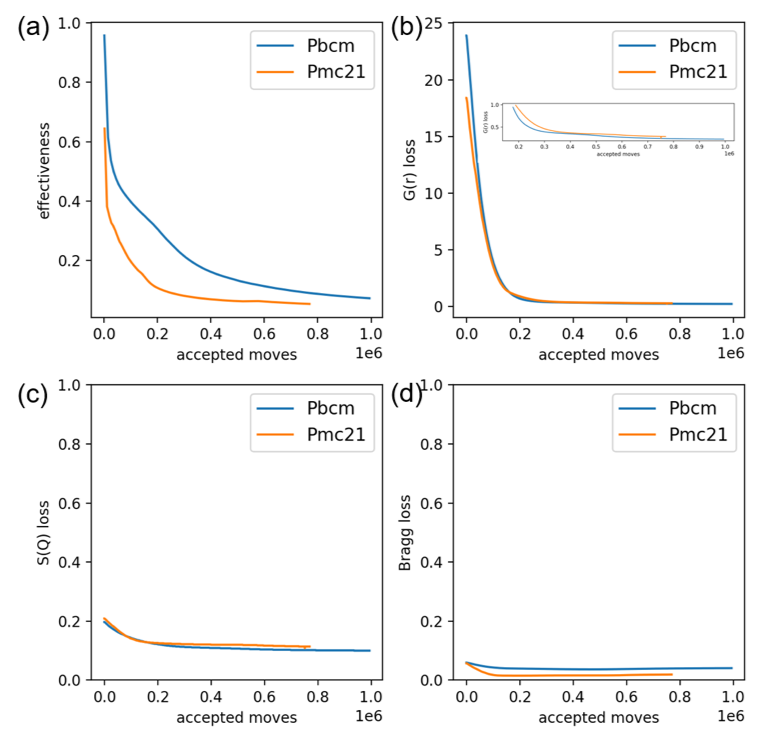

Supplement: Supplementary Materials — Figure S1: PDFs of AN (500 K). Figure S2: PDF refinements for ANL4. Figure S3: Lattice parameter cp as a function of La content. Figure S4: a comparison of the fitting quality between calculations with starting model of Pbcm and Pmc21 space groups. Figure S5: results of data fitting for AN and ANL4 at 300 K. Figure S6: illustration of the projection of Ag displacements. Figure S7: projection of the <001>c displacement of Ag. Figure S8: overall view of the <001>c Nb atom displacement. Figure S9: overall view of the <001>c Ag atom displacement. Figure S10: temperature-dependent dielectric constant of AN, ANL2, and ANL4. Figure S11: RMC fitting results for the verification test. Figure S12: analysis for the RMC verification test. [file 9782343.f1.zip › S4.png]

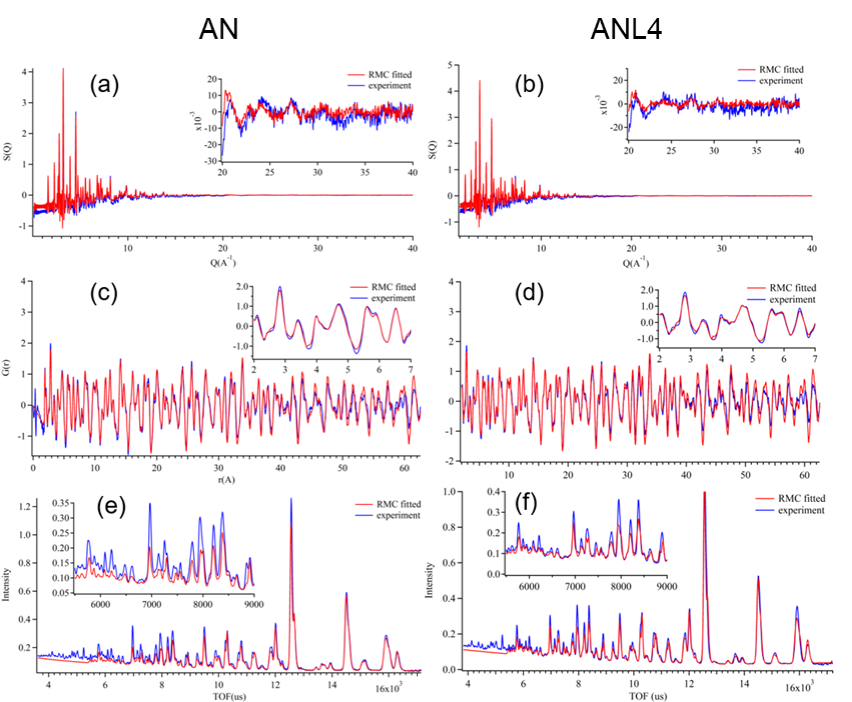

Supplement: Supplementary Materials — Figure S1: PDFs of AN (500 K). Figure S2: PDF refinements for ANL4. Figure S3: Lattice parameter cp as a function of La content. Figure S4: a comparison of the fitting quality between calculations with starting model of Pbcm and Pmc21 space groups. Figure S5: results of data fitting for AN and ANL4 at 300 K. Figure S6: illustration of the projection of Ag displacements. Figure S7: projection of the <001>c displacement of Ag. Figure S8: overall view of the <001>c Nb atom displacement. Figure S9: overall view of the <001>c Ag atom displacement. Figure S10: temperature-dependent dielectric constant of AN, ANL2, and ANL4. Figure S11: RMC fitting results for the verification test. Figure S12: analysis for the RMC verification test. [file 9782343.f1.zip › S5.png]

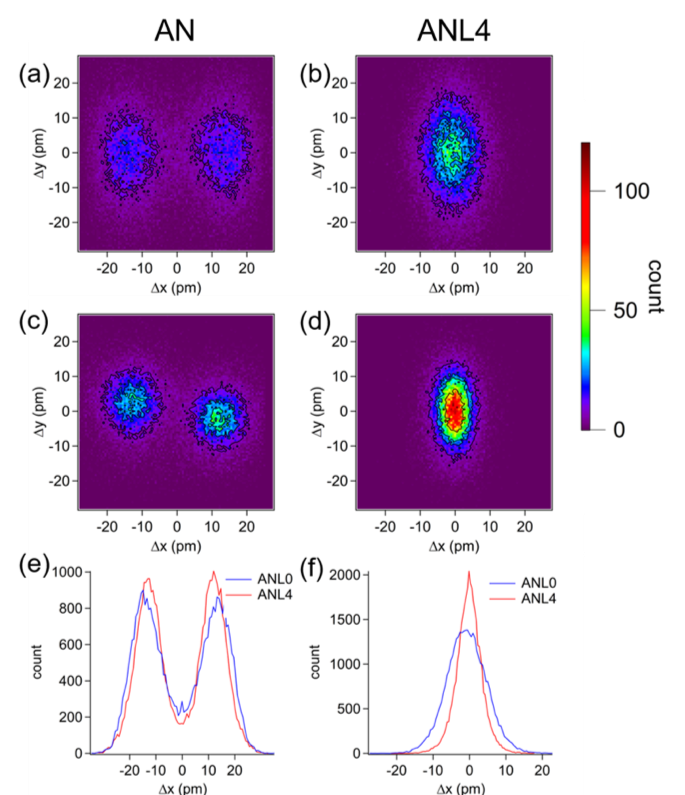

Supplement: Supplementary Materials — Figure S1: PDFs of AN (500 K). Figure S2: PDF refinements for ANL4. Figure S3: Lattice parameter cp as a function of La content. Figure S4: a comparison of the fitting quality between calculations with starting model of Pbcm and Pmc21 space groups. Figure S5: results of data fitting for AN and ANL4 at 300 K. Figure S6: illustration of the projection of Ag displacements. Figure S7: projection of the <001>c displacement of Ag. Figure S8: overall view of the <001>c Nb atom displacement. Figure S9: overall view of the <001>c Ag atom displacement. Figure S10: temperature-dependent dielectric constant of AN, ANL2, and ANL4. Figure S11: RMC fitting results for the verification test. Figure S12: analysis for the RMC verification test. [file 9782343.f1.zip › S6.png]

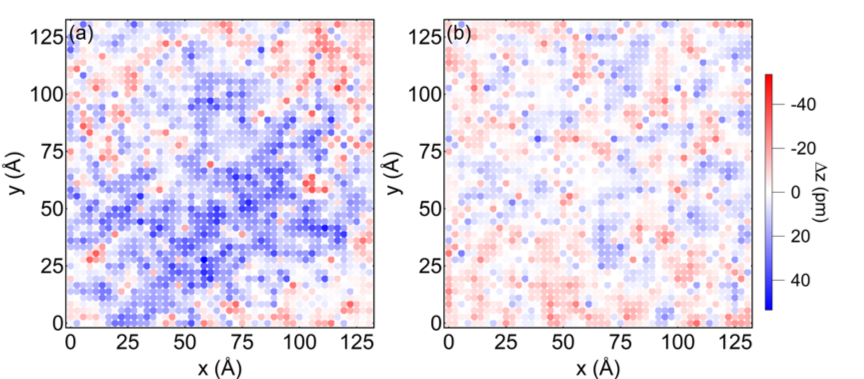

Supplement: Supplementary Materials — Figure S1: PDFs of AN (500 K). Figure S2: PDF refinements for ANL4. Figure S3: Lattice parameter cp as a function of La content. Figure S4: a comparison of the fitting quality between calculations with starting model of Pbcm and Pmc21 space groups. Figure S5: results of data fitting for AN and ANL4 at 300 K. Figure S6: illustration of the projection of Ag displacements. Figure S7: projection of the <001>c displacement of Ag. Figure S8: overall view of the <001>c Nb atom displacement. Figure S9: overall view of the <001>c Ag atom displacement. Figure S10: temperature-dependent dielectric constant of AN, ANL2, and ANL4. Figure S11: RMC fitting results for the verification test. Figure S12: analysis for the RMC verification test. [file 9782343.f1.zip › S7.png]

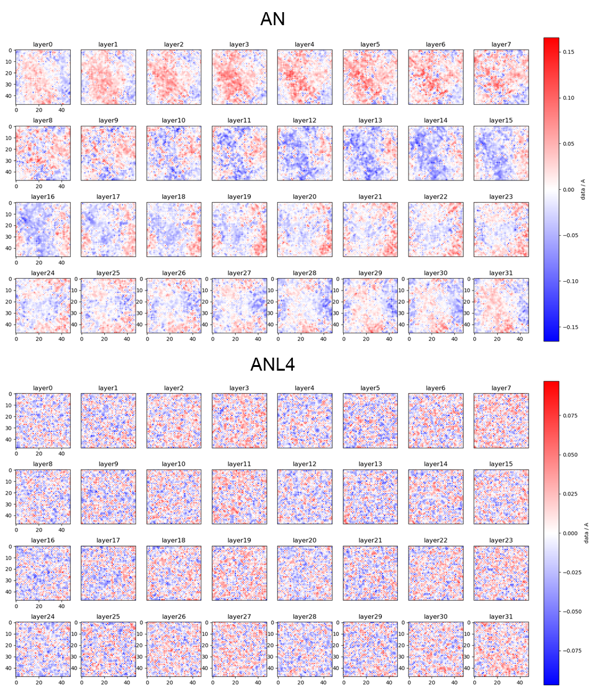

Supplement: Supplementary Materials — Figure S1: PDFs of AN (500 K). Figure S2: PDF refinements for ANL4. Figure S3: Lattice parameter cp as a function of La content. Figure S4: a comparison of the fitting quality between calculations with starting model of Pbcm and Pmc21 space groups. Figure S5: results of data fitting for AN and ANL4 at 300 K. Figure S6: illustration of the projection of Ag displacements. Figure S7: projection of the <001>c displacement of Ag. Figure S8: overall view of the <001>c Nb atom displacement. Figure S9: overall view of the <001>c Ag atom displacement. Figure S10: temperature-dependent dielectric constant of AN, ANL2, and ANL4. Figure S11: RMC fitting results for the verification test. Figure S12: analysis for the RMC verification test. [file 9782343.f1.zip › S8.png]

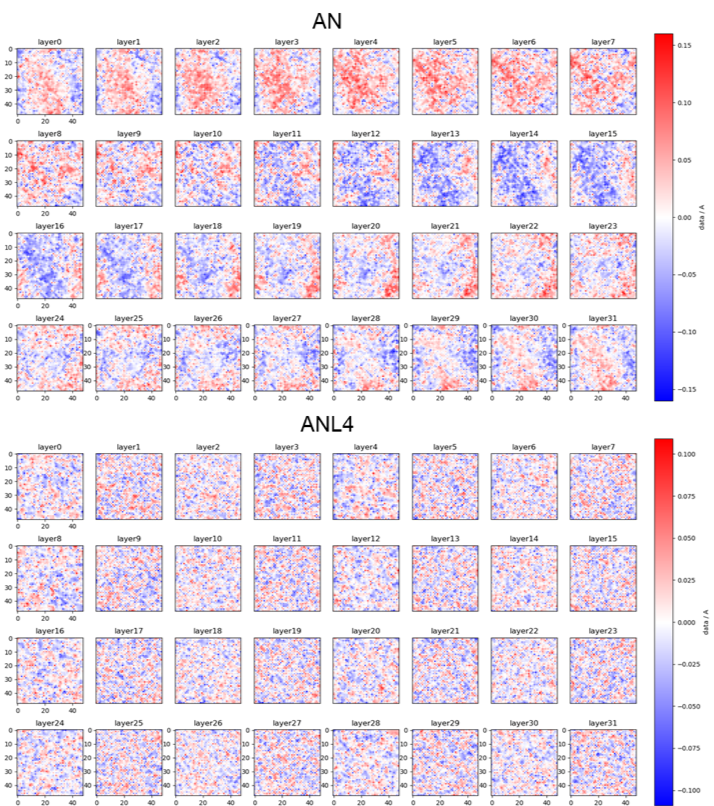

Supplement: Supplementary Materials — Figure S1: PDFs of AN (500 K). Figure S2: PDF refinements for ANL4. Figure S3: Lattice parameter cp as a function of La content. Figure S4: a comparison of the fitting quality between calculations with starting model of Pbcm and Pmc21 space groups. Figure S5: results of data fitting for AN and ANL4 at 300 K. Figure S6: illustration of the projection of Ag displacements. Figure S7: projection of the <001>c displacement of Ag. Figure S8: overall view of the <001>c Nb atom displacement. Figure S9: overall view of the <001>c Ag atom displacement. Figure S10: temperature-dependent dielectric constant of AN, ANL2, and ANL4. Figure S11: RMC fitting results for the verification test. Figure S12: analysis for the RMC verification test. [file 9782343.f1.zip › S9.png]
